# CDMAP/CDVIS: context-dependent mutation analysis package and visualization software

**DOI:** 10.1093/g3journal/jkac299

**Published:** 2022-12-12

**Authors:** David L Patton, Thomas Cardenas, Perrin Mele, Jon Navarro, Way Sung

**Affiliations:** Department of Bioinformatics and Genomics, University of North Carolina at Charlotte, 9201 University City Boulevard, Charlotte, NC, 28223, USA; Department of Bioinformatics and Genomics, University of North Carolina at Charlotte, 9201 University City Boulevard, Charlotte, NC, 28223, USA; Department of Bioinformatics and Genomics, University of North Carolina at Charlotte, 9201 University City Boulevard, Charlotte, NC, 28223, USA; Department of Bioinformatics and Genomics, University of North Carolina at Charlotte, 9201 University City Boulevard, Charlotte, NC, 28223, USA; Department of Bioinformatics and Genomics, University of North Carolina at Charlotte, 9201 University City Boulevard, Charlotte, NC, 28223, USA

**Keywords:** context-dependent mutations, software package, R software, interactive visualization tool, mutation accumulation

## Abstract

The Context-dependent Mutation Analysis Package and Visualization Software (CDMAP/CDVIS) is an automated, modular toolkit used for the analysis and visualization of context-dependent mutation patterns (site-specific variation in mutation rate from neighboring-nucleotide effects). The CDMAP computes context-dependent mutation rates using a Variant Call File (VCF), Genbank file, and reference genome and can generate high-resolution figures to analyze variation in mutation rate across spatiotemporal scales. This algorithm has been benchmarked against mutation accumulation data but can also be used to calculate context-dependent mutation rates for polymorphism or closely related species as long as the input requirements are met. Output from CDMAP can be integrated into CDVIS, an interactive database for visualizing mutation patterns across multiple taxa simultaneously.

## Introduction

Mutations are a primary source of genetic variation and understanding how, where, and when mutations arise is critical to elucidating the evolutionary process. Studying the rate and spectrum of spontaneous mutations can provide insight into how genomes evolve and adapt to changing environments. Spontaneous mutations are known to vary in size, scope, and type, with base substitutions and insertion–deletion mutations ranging from a single nucleotide to several thousand kilobases, and in some cases, entire chromosomes (Baer *et al*. 2007; Gordo *et al*. 2011; Heilbron *et al*. 2014; Lee *et al.* 2012; Sung *et al*. 2015; Sung *et al.* 2016; Keith *et al*. 2016; Wei *et al*. 2018).

Mutation accumulation (MA) studies, where organisms are bottlenecked to accumulate all but the most deleterious mutations (Dillon *et al*. 2015; Long *et al*. 2015, 2018; Lynch *et al*. 2016; Senra *et al.* 2018; Sun *et al.* 2017), have provided a wealth of information regarding how organisms mutate. However, these data have also shown that mutation rate varies depending on the genomic position (Foster *et al*. 2013, Dillon *et al.* 2017), replication strand (Sung *et al*. 2015), mutation type (Long *et al*. 2014), and genomic context (Long *et al*. 2014; Schroeder *et al*. 2016; Harris and Pritchard 2017). Local sequence context has been shown to influence site-specific mutation rates by up to 75-fold within the same sequence context (Dillon *et al*. 2015; Sung *et al*. 2015) and upwards of 403-fold within different contexts (Schroeder *et al*. 2016). Local sequence context has also been shown to have a large impact on site-specific mutation rates in bacteria, plants, and humans (Morton *et al*. 2006; Harris and Nielsen 2014; Harris 2015).

Although evidence of context-specific mutation patterns has been observed across taxonomical life, our understanding of these patterns remains limited due to the ad hoc methods employed in various studies that are designed specifically for a single organism (Lee *et al*. 2016; Long *et al*. 2014; Dillon *et al*. 2018). Furthermore, these studies do not orient the mutations with respect to any genomic landmark (e.g. origin of replication) so it is nearly impossible to examine and contrast large-scale patterns driving spatiotemporal variation in mutation rate across multiple taxa.

To this extent, we have developed CDMAP, an analysis and visualization package to measure the genome-wide rate and spectra of context-dependent mutations. CDMAP is a novel software package that can be used to categorize mutations and their local sequence context into a per-replichore or per-chromosome basis, generate estimates of context-dependent mutation rates, provide a graphical representation and statistical correlation of these rates to compare across multiple taxa, and the output data can be integrated into an interactive graphical database via CDVIS. CDMAP and CDVIS provide a new toolset written in the R programming language providing uniform treatment for categorizing context-dependent mutation patterns and providing a comparative platform that has not been previously established. Being able to dissect mutational patterns via visualization tools can illuminate our understanding of the mechanisms driving replication fidelity and genome evolution.

## Methods

**Fig. 1.**
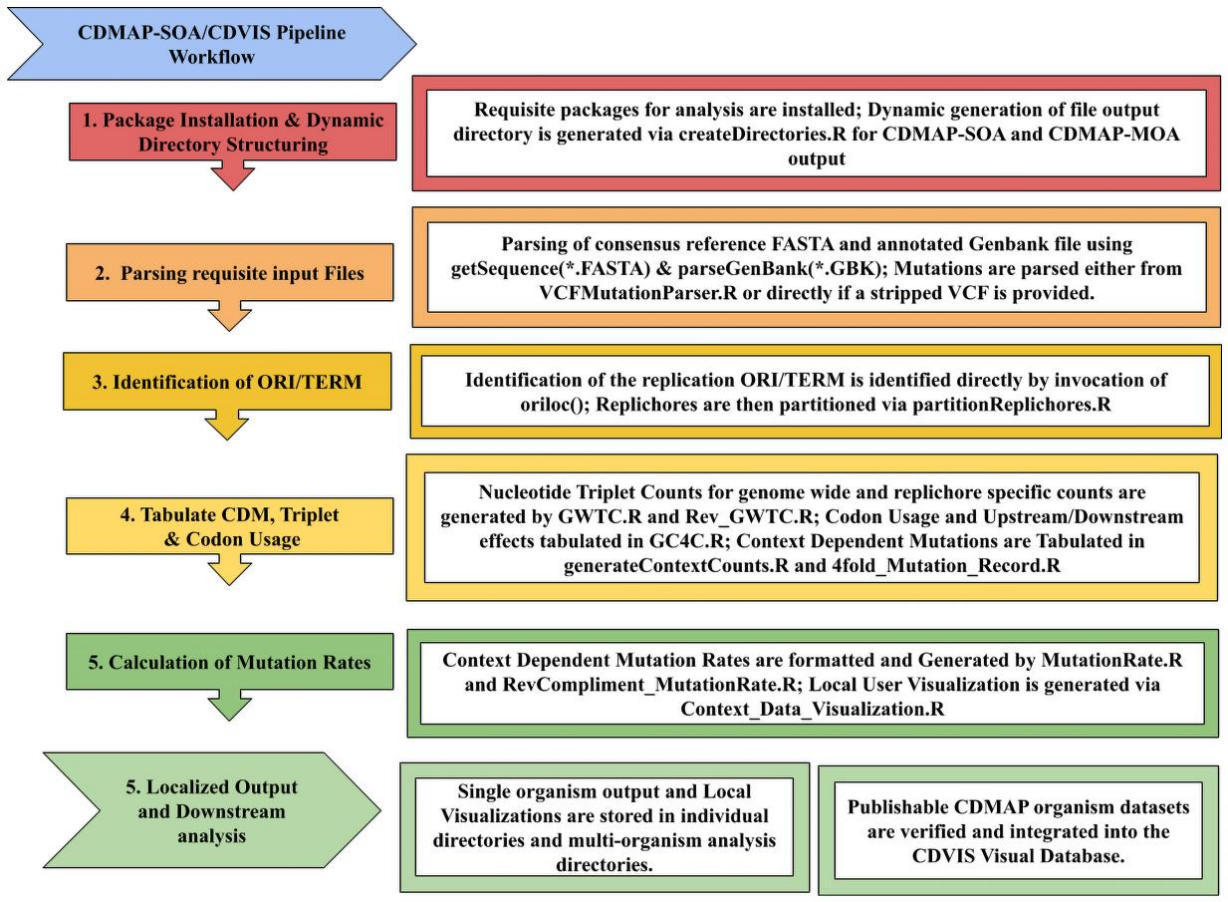
CDMAP/CDVIS diagram workflow. Visual outlining the major steps taken during the analysis of the CDMAP-SOA pipeline for generation of context-dependent mutation rates, genome-wide triplet and codon usage counts on a per-chromosome or per-replichore basis.

The functionality of CDMAP and CDVIS are broken into three separate components of analysis (Fig. 1). The first is the CDMAP Single Organism Analysis pipeline (SOA). The SOA pipeline provides the backbone analysis that catalogs nucleotide motifs across the genome, calculates context-dependent mutation rates, and provides output csv files and organism-specific visualization output via Lattice (Sarkar 2008). The second component, CDMAP Multi-Organism Analysis Pipeline (CDMAP-MOA), generates statistical correlations between different SOA analyses outlining potential relationships in contextual mutation patterns. The final component, CDVIS interfaces with CDMAP output to provide an accessible database of spatiotemporal variation in mutation patterns across analyzed genomes.

### Dependencies and required input files

To identify context-dependent mutation patterns, we use the R programming language, which is a robust library of bioinformatics, statistical, and data visualization packages. Several R dependencies are required for data preprocessing and postprocessing:

- SeqInR (OriLoc): R packages used to parse FASTA sequences and identify the origin of replication for strand-specific analyses (Charif and Lobry 2007)
- Pracma: Numerical and statistical algorithms (Borchers 2021)
- Genbankr: Genbank file parsing (Becker and Lawrence 2019)
- Lattice: Lightweight data visualization package (Sarkar 2008)

Necessary input data for CDMAP includes a modified Variant Call File (VCF), the reference FASTA file, and an annotated Genbank file (GBK) file. A VCF is a space or tab-delimited file that can be generated by variant calling pipelines e.g. (SAMTOOLS/GATK) (Li *et al*. 2009; Van der Auwera and O’Connor, 2020), containing the nucleotide position, the reference nucleotide, and the mutant nucleotide. The reference FASTA file is the genome sequence of the organism that corresponds to the nucleotide positions found in the VCF file. The annotated GBK contains information about the location of genes in the reference FASTA. The reference FASTA, VCF, and GBK files used in the development of this package can be downloaded from the National Center for Biotechnology Information (NCBI) and the Sequence Read Archive at NCBI.

### Replication origin determination and replichore partitioning

Context-specific mutation patterns have been shown to be asymmetrical with respect to the origin of replication (ORI) and replication terminus (TERM), such that the upstream 5′ and downstream 3′ base from the mutant site can influence site-specific mutation rate (Lee *et al*. 2012; Sung *et al*. 2015). CDMAP orients each mutation to a user-defined ORI location or an ORI defined by the OriLoc dependency (Frank and Lobry 2000), an R package used to determine the minimum and maximum cumulative composite skew at synonymous sites (GC skew) that is widely used to identify the ORI in bacterial organisms. CDMAP orients all variants with respect to the ORI for downstream analysis. After successful orientation and partitioning of the sequence data and mutations with respect to their ORI and TERM, genome-wide triplet counts (GWTCs) for the chromosome and each replichore are tabulated for subsequent calculations.

### Nucleotide and mutation frequency determination and rate analysis for mutation accumulation

To calculate the context-dependent mutation rate for MA experiments, genome-wide and replichore-wide triplet counts are counted (GWTC/RWTC). CDMAP then parses the VCF to determine the upstream and downstream nucleotide associated with each variant and computes the mutation frequency and the context-dependent mutation rate at all 64 possible triplets:


$$U_{bs}=\;\frac{M_{triplet}}{\left(GWTC_{triplet})(G)(N\right)}\;R_{bs}=\;\frac{M_{triplet}}{\left(RWTC_{triplet})(G)(N\right)}$$

Thecontext-dependent mutation rate for a triplet in the chromosome (*U*_*bs*_) is then determined by the total number of mutations observed at the center nucleotide of that triplet (*M*_*triplet*_) divided by the triplet count for the genome (*GWTC*_*triplet*_) the number of lineages (*N*) and the estimated number of generations elapsed (*G*). Replichore-specific rates are similarly calculated using mutations observed in a replichore (*R*_*bs*_) divided by the triplet count for that replichore (*RWTC*_*triplet*_), *G*, and *N*. In addition to a triplet frame, CDMAP accounts for and tracks data regarding upstream and downstream neighboring nucleotides in a 5-mer reference frame, i.e. NXNN downstream and NNXN nucleotide upstream contexts, where X is the mutable nucleotides, and N represents further upstream and downstream nucleotides from X. The following analysis in this paper focuses on context-dependent mutation rates at triplets, but we have added the functionality for additional neighboring sites based on findings that upstream and downstream sites exceeding the immediate local nucleotides have an effect on the site-specific mutation rate in plants (Morton 2022) and humans (Aggarwala and Voight 2016; Zhu *et al*. 2017; Simon and Huttley 2020).

### Multi-organism analysis

CDMAP was developed to allow for flexibility in the number of organisms analyzed. During the run process for a single organism, CDMAP dynamically creates a repository of the output which can be used for downstream comparison against additional CDMAP runs. Once selected genomes have been analyzed, the user can perform a multi-organism analysis to compare the context-dependent mutation patterns generated using the SOA pipeline. This comparison can occur on a chromosome-wide, strand-specific, or replichore-specific basis. CDMAP performs a one-to-many Pearson's correlation sequentially with each organism and automatically orients the coefficients according to GC content for display as heat maps in the lattice.

### CDMAP-SOA visualization

**Fig. 2.**
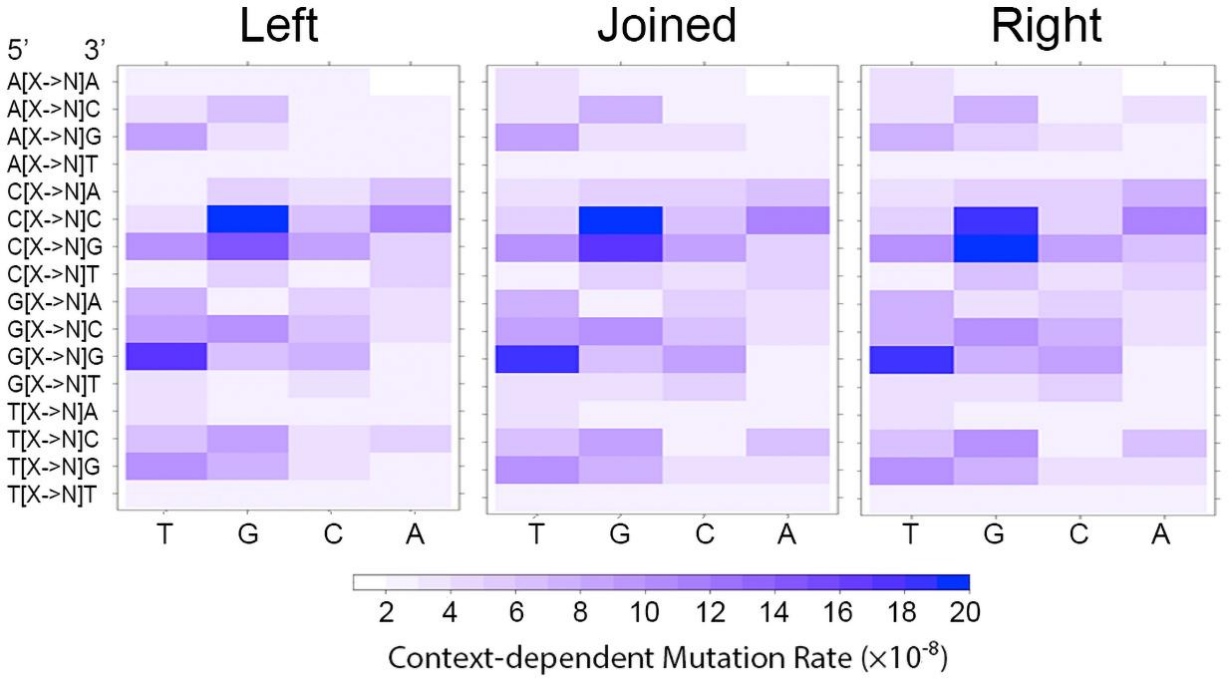
CDMAP-SOA output of Bacillus subtilis mismatch repair deficient MA lines. Context-dependent mutation rates shown for left replichore and right replichore. Each row repesents a mutation at a triplet N1[X->N2]N3 with X -> N2 repesenting the reference nucleotide (shown on the X-axis) and mutation to any other nucleotide surrounded by two nucleotides N1 and N3. Contexts in the right replichore are handled as reverse complement to match was done in Sung et al., 2015.

Complex patterns within large-scale data sets are often easier to identify using visualization tools. Relevant information about triplet frequency, variant distribution, and genome-wide and replichore-specific mutation rates are passed through Lattice, and correlation between input files can be automatically formatted (Fig. 2) (Sarkar 2008). Throughout the process, CDMAP collects and outputs both CSV format spreadsheets and heatmaps in dynamically generated output directories that are categorized for easy navigation and downstream analyses.

In the example shown in Fig. 2, CDMAP has generated the context-dependent mutation rates for all 64 nucleotide triplets from a mismatch repair deficient line of *B. subtilis* (Sung *et al*. 2015). In Fig. 2, site-specific rates are shown for the left and right replichores, with each context-oriented so that both strands are synthesized identically (mutations and contexts are taken with respect to their reverse complement for the right replichore). The standard reference sequence is displayed from 5′ -> 3′, and this would then make the right replichore the lagging strand template and the left replichore the leading-strand template.

As DNA is double-stranded, the complementary strand of the reference sequence would then be synthesized in the reverse direction. To allow for context analyses in any orientation, CDMAP generates context data for both the reference strand and the complementary strand. This feature allows for the contrast of strand-specific errors that arise on either the leading or lagging strand. When we apply our algorithm to generate context-dependent mutation rates in *Bacillus subtilis* MA lines, we are able to recapitulate similar results to ad hoc methods used previously (Sung *et al*. 2015), whereby the contexts in the right replichore are handled in the reverse complement (Fig. 2). We note that while most of the rates remain consistent regardless of how the replichores are handled, there are slight differences in the number of mutations in each replichore when using a more precise ORI and TER generated from OriLoc (Supplementary Table 2).

### CDMAP-MOA visualization

**Fig. 3.**
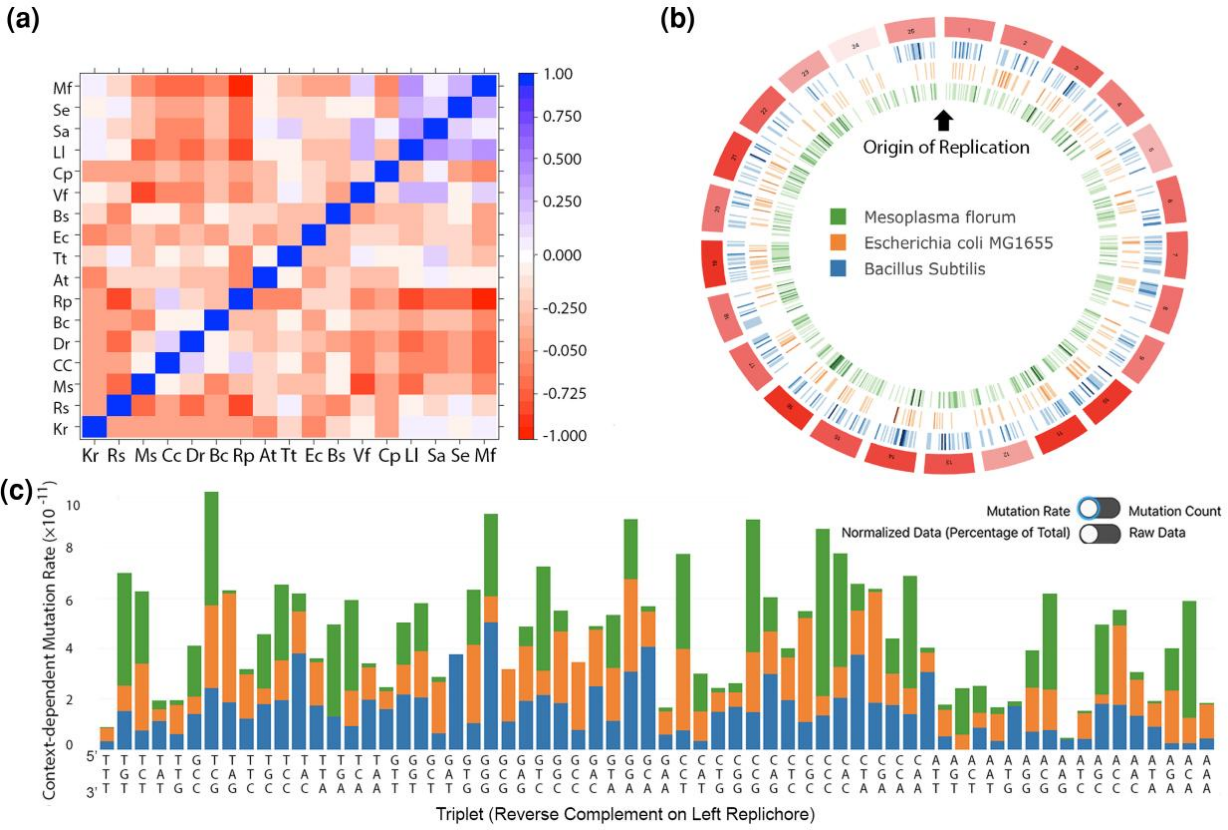
CDMAP/CDVIS Webtool. A) CDMAP One-to-Many Correlation Heatmap for the primary chromosome of 17 bacterial mutation accumulation data sets (Pearson's correlation coefficient sorted by coding region GC content—Supplementary Table 1). B) CDVIS online CIRCOS (Krzywinski et al. 2009) visualization of *B. subtilis, E. coli, and M. florum*. Mutations for each organism are oriented into 25 bins with the origin of replication located at bin 1 and the terminus located in bin 13/14. Intensity of tick marks show the number of mutations for that species, and intensity of boxes indicate increasing density of mutations across selected organisms. Visualization tools can display cumulative mutation data across multiple organisms for each selected bin (www.wsunglab.com:3000). C) Sixty-four codon triplet stacked graph indicating the context-dependent mutation rate of *B. subtilis, E. coli, and M. florum* and can be displayed as a rate, raw counts, or conditional rate per triplet in CDVIS.

In Fig. 3a, we show an example of multiple organisms benchmarked via CDMAP-SOA (Supplementary Table 1). In this visualization, rows are ranked from AT-rich (top) to GC-rich (bottom) and columns are oriented GC-rich (left) to AT-rich (right). The one-to-one comparison of context-dependent mutation rates between organisms are color-coordinated relative to Pearson's coefficient, as indicated by the heat map legend (Fig. 3a).

### Web visualization using CDVIS

Data generated through the CDMAP pipeline can be integrated into our front-facing web server, the Context-dependent Visualization Software (CDVIS). CDVIS contains CDMAP output from existing MA experiments that can be used as a comparative framework against future data sets. CDVIS takes the output from the CDMAP pipeline and organizes them into JSON objects that are then dynamically loaded into a circular format using CIRCOS (Krzywinski *et al*., 2009) (Fig. 3b).

The user can select numerous datasets from available pre-loaded MA experiments to visualize the spatiotemporal variation of mutation rate in MA organisms (www.wsunglab.com:3000). Each circular track represents a single organism, and tick marks within the tracks indicate the location and density of mutations at that location. The genome is divided into selectable bins (size 25/50/75) and the density/type/and rate of mutations of the selected organism(s) are displayed in a side panel. For easy comparison, CDMAP/CDVIS displays the first bin starting at the origin of replication. Finally, the visualization tools allow for the summation of conditional mutation rates (mutation rates normalized to genome-wide nucleotide content—Fig. 3b) and cumulative context-dependent mutation rates for different triplets (Fig. 3c). At this time, additions to the available visualized organisms can be made through email requests to the authors.

### Results and discussion

CDMAP was quantitatively benchmarked against 17 MA data sets in prokaryotic organisms (Kibota and Lynch 1996; Sung *et al*. 2012, 2015; Long *et al*. 2014; Dillon *et al*. 2015; Gilchrist *et al*. 2015; Lynch *et al*. 2016; Kucukyildirim *et al*. 2016), which harbor a variety of different genomic architectural features (Supplementary Table 1). The majority of MA studies contain organisms with a singular, circular chromosome such as *Escherichia coli*, while others may have multiple genomic elements such as chromids and plasmids (Supplementary Table 1), or may be deficient in repair enzymes such as mismatch repair. We processed 17 organisms (Supplementary Table 1) containing a total of 12493 mutations. There are no other software toolkits that are readily available for comparison, but we were able to recover identical context-dependent mutation rates from prior studies generated using ad hoc methods (Lee *et al* 2012; Sung *et al*. 2015). All data were uploaded to the CDVIS visualization tool at www.wsunglab.com:3000.

It is important to note that CDMAP operates on a one-to-one basis, i.e. the SOA pipeline only analyzes one set of mutational variants from a VCF relative to a reference FASTA and GBK file at a time. If a user wishes to compare multiple substrain variants against a single reference, then multiple runs of CDMAP will need to be conducted to account for each substrain analyzed. Although this program has been benchmarked using MA lines, CDMAP can be also used to compare two closely related strains, with one strain designated as the reference and one strain designated as the derived strain, as long as the input requirements are met. This feature could be used to contrast context-dependent mutation processes between two closely related species or natural isolates from a population.

CDMAP was designed to be a lightweight analysis package capable of running on a standard laptop or desktop. Each of the 17 data sets were analyzed on an iMac with a 2.9 Ghz quad core intel i5 processor, 16GB 1600Mhz DDR3 ram, and running MAC OSX Catalina. On the benchmarked machine, CDMAP utilized ∼1GB (6.25% memory, 7 threads) and roughly 60% CPU utilization during its most computationally intensive processes. The average runtime of a given organism came in around 90 min for an average-size bacterial genome (∼5Mb).

### Practical example

The following commands can be used as a practical example of how CDMAP can be used to generate and analyze context-dependent mutation patterns in genomic data. In this short tutorial, we will walk through basic commands used to generate context-dependent mutation patterns from a *Bacillus subtilis* MA dataset (Sung *et al*. 2015). This example data for *B. subtilis* and other organisms used for benchmarking are included with CDMAP package found on the Github repository found at (https://github.com/DLP-Informatics/CDMAP).

CDMAP by default should install all of the necessary R packages when running CDMAP_SingleOrganismAnalysis.R for the first time; however, if you wish to install these prior to your first run, the following packages are necessary to run CDMAP (note: your machine may require administrative privileges to install these packages):

- SeqInR
- BiocManager
- Pracma
- Beepr
- Lattice
- Tidyverse
- vcfR
- stringr
- genbankr (contained in BiocManager)

To begin running CDMAP, first install R (https://www.r-project.org/), then navigate to the directory in which you unpacked the CDMAP package and execute the following command in terminal:

>*Rscript CDMAP_SingleOrganismAnalysis.R*

The user will be prompted for the name of the output folder designated by the end user, whether the user is using a VCF or modified base call file, and the full path location of the reference sequence, genbank file, and the VCF or modified base call file.

>*Bacillus_subtilis_WT*
>*basecall*
>*/Users/Username/Desktop/CDMAP/Test_Datasets/bacillus/Bacillus_3610.fasta*
>*/Users/Username/Desktop/CDMAP/Test_Datasets/bacillus/NC000964.gbk*
>*/Users/Username/Desktop/CDMAP/Test_Datasets/bacillus/Bacillus_WT.csv*

CDMAP will then prompt the user for how many generations have elapsed and how many lineages were in the experiment. For analyzing data that is not from a MA experiment, generations can be estimated using a molecular clock method. By default, mutation rates will be scaled to 1 × 10^−8^ for ease of visualization in the lattice, but scaling can be changed by the user (0 for default, 1 for scaling to the average mean of rates, or 2 for custom parameters). Finally, the user will be prompted for manual input (yes/no) of the ORI and TER or it will be determined automatically using OriLoc. If the user uses OriLoc to determine the ORI, it will also use OriLoc to determine the optimal TER position, otherwise, the user must manually specify the replication ORI and TER position for CDMAP. For example, the *B. subtilis* wild-type MA experiment underwent 5077 generations across 50 lines, and when we are prompted, we input yes when prompted and manually input the ORI and TER.

>*5077*
>*50*
>*0*
>yes **no to use OriLoc
*0*
*2107299*

After these following steps have been completed, CDMAP-SOA will have the requisite information needed to count contexts and automatically estimate mutation rates for the chromosome, for each replichore (on both strands), and generate high-resolution heatmaps that can be accessed by a lattice (Fig. 2). Once the run is complete, all SOA output files will be placed into the CDMAP_Output/Output_Directory that was designated by the user. Upon request, these data can be interfaced into CDVIS for further analysis. If the user wants to perform a direct correlation between the rates from different organisms or experiments, they can invoke CDMAP-MOA using the following command:

*Rscript CDMAP_MultiOrganism_Analysis.R*

CDMAP-MOA will perform a Pearson's correlation between the context-dependent mutation rates for all SOA runs located within the specified Output_Directory, sort the organisms by GC content, and generate a high-resolution correlation heatmap for downstream analysis (Fig. 3a). An in-depth description of the files generated, information on the directory structure, and a full technical document can be found in the CDMAP technical document which can be found in the package, as well as at the github repository (https://github.com/DLP-Informatics/CDMAP/blob/main/Documentation/CDMAP_Technical_Documentation.docx).

### Conclusion

CDMAP is a toolkit designed to streamline the analysis of context-dependent mutations from genomic sequence data. While CDMAP has been benchmarked on bacterial MA data sets with a single replication origin, CDMAP is capable of analyzing linear chromosomes, including viral, archaeal, and eukaryotic data sets with the caveat of manually inputting the ORI. Determining the ORI in nonprokaryotic chromosomes can be done in a few different ways, including replication profile construction via deep sequencing methods (Xu *et al*. 2012). In addition, CDMAP can not only be applied to mutation datasets but also to silent sites from population sequencing. The application of CDMAP on data from natural populations and integration into CDVIS can assist researchers in exploring how spatiotemporal variation in mutation rate can drive genome evolution.

## Supplementary Material

jkac299_Supplementary_Data

## Data Availability

The CDMAP source code is freely available for noncommercial academic use at https://github.com/DLP-Informatics/CDMAP; genomic data can be accessed at NCBI from the Accession numbers in Supplementary Table 1. Visualization data are also available at CDVIS for viewing at: wsunglab.com:3000 Supplemental material is available at *G3* online.
