# Supplementary material for: CDMAP/CDVIS: context-dependent mutation analysis package and visualization software
Source: G3 (Bethesda). 2022 Dec 12;13(4):jkac299. doi: 10.1093/g3journal/jkac299 (PMC10085751; doi:10.1093/g3journal/jkac299)
Supplement: jkac299_Supplementary_Data [file jkac299_supplementary_data.zip › Table_S1_G3-2022-403638.docx]

| Species | Acc #. GCF/GCA: | Gen. | Lines | Chr. | Mut. | GC% | ORI (KB) |
| --- | --- | --- | --- | --- | --- | --- | --- |
| *Agrobacterium tumefaciens C58* | 000092025.1_ASM9202v1 | 5819 | 47 | 2 | 233 | 60 | 2765 |
| *Bacillus subtilis NCIB 3610* | 000186085.1_ASM18608v1 | 5077 | 50 | 1 | 350 | 44 | 4170 |
| *Bacillus subtilis NCIB 3610 (MMR-)* | 000186085.1_ASM18608v1 | 5077 | 50 | 1 | 5295 | 44 | 4170 |
| *Burkholderia cenocepacia HI2424* | 000203955.1_ASM20395v1 | 5554 | 47 | 3 | 130 | 67 | 69 |
| *Caulobacter crescentus NA1000* | 000022005.1_ASM2200v1 | 4284 | 44 | 1 | 259 | 68 | 3818 |
| *Colwellia psychrerythraea 34H* | 000012325.1_ASM1232v1 | 1078 | 84 | 1 | 400 | 39 | 4903 |
| *Deinococcus radiodurans BAA-816* | 001638825.1_ASM163882v1 | 5961 | 43 | 2 | 331 | 68 | 258 |
| *Escherichia coli K-12 MG1655* | 000005845.2_ASM584v2 | 1682 | 46 | 1 | 1623 | 52 | 3644 |
| *Escherichia coli K-12 MG1655 (MMR -)* | 000005845.2_ASM584v2 | 1682 | 46 | 1 | 231 | 52 | 3644 |
| *Kineococcus radiotolerans SRS30216* | 000017305.1_ASM1730v1 | 4724 | 44 | 1 | 280 | 74 | 522 |
| *Lactococcus lactis DSMZ20481* | 900088425.1_A12 | 3973 | 63 | 1 | 813 | 37 | 1 |
| *Mesoplasma florum L1* | 000479355.1_ASM47935v1 | 2351 | 28 | 1 | 544 | 27 | 326 |
| *Mycobacterium smegmatis MC2 155* | 000283295.1_ASM28329v1 | 4900 | 49 | 1 | 856 | 68 | 3609 |
| *Rhodobacter sphaeroides ATCC 17025* | 000016405.1_ASM1640v1 | 4544 | 46 | 1 | 107 | 69 | 2 |
| *Ruegeria pomeryoi DSS-3* | 000011965.2_ASM1196v2 | 5386 | 47 | 1 | 147 | 65 | 1 |
| *Staphyloccus aureus ATCC 25923* | 000756205.1_ASM75620v1 | 2716 | 83 | 1 | 274 | 34 | 3 |
| *Staphyloccus epidermis ATCC 122228* | 000007645.1_ASM764v1 | 7101 | 22 | 1 | 294 | 33 | 2419 |
| *Teredinibacter turnerae T7901* | 000023025.1_ASM2302v1 | 3025 | 42 | 1 | 779 | 52 | 4252 |
| *Vibrio fischeri ES114* | 000011805.1_ASM1180v1 | 5187 | 48 | 2 | 132 | 40 | 1 |
| *Vibrio fischeri ES114 (MMR -)* | 000011805.1_ASM1180v1 | 5187 | 48 | 2 | 2909 | 40 | 1 |
|  | | | | | | | |
| Table S1 - Mutation accumulation datasets used during the development of CDMAP, and generated visualization data can be accessed using CDVIS (www.wsunglab.com:3000). Table headers are species name, accession GCF or GCA number of the reference genome assembly used in the analysis (FASTA/GBK files), # of MA generations, # of MA lineages, chromosomes, total observed mutations, GC-content and the kilo base location of the origin of replication (ORI). MMR- indicates mismatch-repair deficient strain(Senra et al. 2018; Dillon et al. 2017; Sun et al. 2017; Kucukyildirim et al. 2016a; Sung et al. 2016; Kucukyildirim et al. 2016b; Lee et al. 2016; Sung et al. 2015; Long et al. 2015a; Sung et al. 2012b; Long et al. 2018; Dillon et al. 2015). | | | | | | | |
|  |  |  |  |  |  |  |  |
|  |  |  |  |  |  |  |  |
|  |  |  |  |  |  |  |  |
