# Supplementary material for: CDMAP/CDVIS: context-dependent mutation analysis package and visualization software
Source: G3 (Bethesda). 2022 Dec 12;13(4):jkac299. doi: 10.1093/g3journal/jkac299 (PMC10085751; doi:10.1093/g3journal/jkac299)
Supplement: jkac299_Supplementary_Data [file jkac299_supplementary_data.zip › Table_S2_G3-2022-403638.docx]

|  |  | ORI set to 0 | | | |  | ORI set using OriLoc | | | |  | | Difference | | | | |  |
| --- | --- | --- | --- | --- | --- | --- | --- | --- | --- | --- | --- | --- | --- | --- | --- | --- | --- | --- |
| Triplet (5'->3') |  | T | G | C | A |  | T | G | C | A | |  | | T | G | C | A | |
| T[X-->Y]T |  | 7 | 17 | 9 | 46 |  | 7 | 17 | 8 | 41 | |  | | 0 | 0 | 1 | 5 | |
| T[X-->Y]G |  | 161 | 57 | 60 | 17 |  | 154 | 54 | 59 | 17 | |  | | 7 | 3 | 1 | 0 | |
| T[X-->Y]C |  | 60 | 88 | 29 | 96 |  | 64 | 91 | 27 | 93 | |  | | 4 | 3 | 2 | 3 | |
| T[X-->Y]A |  | 29 | 28 | 40 | 55 |  | 27 | 29 | 39 | 57 | |  | | 2 | 1 | 1 | 2 | |
| G[X-->Y]T |  | 29 | 12 | 164 | 85 |  | 29 | 12 | 160 | 82 | |  | | 0 | 0 | 4 | 3 | |
| G[X-->Y]G |  | 152 | 52 | 362 | 50 |  | 158 | 55 | 359 | 51 | |  | | 6 | 3 | 3 | 1 | |
| G[X-->Y]C |  | 42 | 107 | 194 | 114 |  | 44 | 110 | 191 | 112 | |  | | 2 | 3 | 3 | 2 | |
| G[X-->Y]A |  | 68 | 29 | 205 | 186 |  | 71 | 31 | 202 | 182 | |  | | 3 | 2 | 3 | 4 | |
| C[X-->Y]T |  | 12 | 27 | 50 | 212 |  | 12 | 29 | 48 | 213 | |  | | 0 | 2 | 2 | 1 | |
| C[X-->Y]G |  | 107 | 175 | 357 | 234 |  | 110 | 183 | 349 | 231 | |  | | 3 | 8 | 8 | 3 | |
| C[X-->Y]C |  | 20 | 132 | 64 | 275 |  | 19 | 136 | 61 | 269 | |  | | 1 | 4 | 3 | 6 | |
| C[X-->Y]A |  | 20 | 35 | 110 | 266 |  | 20 | 36 | 113 | 273 | |  | | 0 | 1 | 3 | 7 | |
| A[X-->Y]T |  | 18 | 5 | 7 | 19 |  | 19 | 4 | 8 | 18 | |  | | 1 | 1 | 1 | 1 | |
| A[X-->Y]G |  | 97 | 32 | 64 | 29 |  | 96 | 34 | 62 | 29 | |  | | 1 | 2 | 2 | 0 | |
| A[X-->Y]C |  | 45 | 84 | 24 | 44 |  | 48 | 88 | 23 | 44 | |  | | 3 | 4 | 1 | 0 | |
| A[X-->Y]A |  | 24 | 8 | 30 | 17 |  | 29 | 9 | 30 | 17 | |  | | 5 | 1 | 0 | 0 | |

Table S2 - Mutation Positioning Change Based Upon OriLoc. This table shows the changes in the type of contextual mutations (5'->3') when the ORI is set at 0 (Sung et al. 2015) or instead defined by OriLoc. Of the total mutations observed in Bacillus subtilis mismatch-repair deficient mutation accumulation lines (152/5293), 2.87% of the mutations have changed context due to adjustment of ORI and TERM positions using Oriloc. In this example the replication ORI was shifted approximately ~2500 bp, and there was a ~250,000 bp shift in terminus position that accounts difference in context-dependent mutation tabulation.
